# The Physician-Assisted Suicide Pathway in Italy: Ethical Assessment and Safeguard Approaches

**DOI:** 10.1007/s11673-023-10302-2

**Published:** 2023-10-13

**Authors:** Luciana Riva

**Affiliations:** Bioethics Unit, Istituto Superiore di Sanità, Via Giano della Bella 34, 00162 Roma, Italia

**Keywords:** Ethics commitee, Physician-assisted suicide law, Euthanasia, Ethics committees for clinical practice

## Abstract

Although in Italy there is currently no effective law on physician-assisted suicide or euthanasia, Decision No. 242 issued by the Italian Constitutional Court on September 25, 2019 established that an individual who, under specific circumstances, has facilitated the implementation of an independent and freely-formed resolve to commit suicide by another individual is exempt from criminal liability. Following this ruling, some citizens have submitted requests for assisted suicide to the public health system, generating a situation of great uncertainty in the application processes. As a matter of fact, shared and defined procedures are lacking as Decision 242/2019 merely added some principles on which the legislature will have to base its future intervention. This paper analyses the advisory role that the Decision attributes to territorial ethics committees with the aim of stimulating discussions on their role in oversight mechanisms. The proposed conclusion is that the envisaged role does not appear consistent with the functions of these bodies and is ultimately substantially undefined and unjustified.

## Introduction

Physician-assisted suicide (PAS) and euthanasia are widely debated worldwide. Even the very definitions of the two terms vary from one country to another and are ethically controversial (Emanuel, et al. 2016; Mroz, et al. 2021; Friesen 2020). For the purposes of this paper, PAS refers to lethal drugs prescribed or provided by a physician at a patient’s request and that are self-administered by the patient with the goal of ending his or her own life; euthanasia—voluntary, non-voluntary, or involuntary—refers to the deliberate, direct causation of death by a physician (Goligher, et al. 2017).

The topic is highly divisive within the public and professional debate, with diverging opinions on key issues such as the existence of conditions in which autonomously chosen death can be considered a benefit (therefore favouring it would be an act of beneficence), the limits of the legislation, and the role of the physician. With respect to the last point, one ultimate position among opponents is that there is no possible role for physicians in either euthanasia or assisted suicide, that physicians have a duty to care for ailing patients and to never cause any harm and that, given the very fiduciary nature of the patient–physician relationship, these practices cannot be consistent with professional practice (Vizcarrondo 2013; Breitbart 2012; Snyder, Sulmasy, and Mueller 2017). However, despite an overt level of criticism from the medical community, over the past two decades, aid-in-dying practices have become more widespread worldwide and have been integrated into some healthcare systems. Assisted dying is legal in about eighteen jurisdictions in Europe, America, the United States, and Australasia and several nations are drafting or considering new legislation (Mroz 2020). The main medical associations have expressed their opposition over time, in some cases adopting neutral positions due to the need to represent the entire spectrum of opinions of their members (Best 2021; Iacobucci 2021; Piili, et al. 2022). The World Medical Association currently continues to declare its firm opposition to euthanasia and PAS (WMA 2019). Support amongst physicians is lower than amongst the public (Emanuel et al. 2016), and in some countries, such as Belgium, acquiescence by the medical community has been almost mandatory (Bosshard, et al. 2008).

Both supporters and opponents convey an irreducible moral tension at all levels of the transition from problem conceptualization to operationalization. Here, “conceptualization” is taken to mean the process of defining the terms, values involved, and even the subject-matter of legislative deeds. “Operationalization” specifically refers to how institutions and legislators establish the necessary safeguards relating to aid-in-dying practices once they have been permitted. It also involves establishing control criteria to ensure proper implementation of the provisions and prevention of errors and abuses. One of the main objections raised against the legalisation of euthanasia and PAS is precisely that once they have been legalized, the process cannot be monitored effectively, which could result in breaches of the rights of vulnerable patients. These practices raise, even after a law has been implemented, important questions about the correct supervision, systems for data collection, and evaluation of cases (Smets, et al. 2009; Raus, et al. 2021; Riley 2022).

A question debated concerns the patient’s decision-making capacity, although the problem of establishing and conceptualizing the minimum cognitive ability required is not unique to the assisted suicide scenario (Seyfried, et al. 2013; Dembo, et al 2020; Price, et al. 2014; Shaw, et al. 2018).

Existing regulations vary with regard to their terminology, content, and level of detail, but all of them establish substantive and procedural requirements: the former concern the suitability of the patient (e.g. age, medical condition, ability to give consent, etc.), while the latter refer to the procedures for the implementation of the request (e.g. consultation with two independent physicians, reporting to the authorities, etc.).

For example, Dutch law defines “statutory due care criteria” as the obligations that a physician about to perform EAS (Euthanasia/Assisted Suicide) must follow. These consist in verification that the patient’s request is voluntary and well thought out; that the suffering is unbearable and without prospects for improvement; that the patient is well informed of his/her situation and prognosis and that there are no reasonable alternative options for the specific case. Procedural criteria include consultation with at least one independent physician—who must consult with the patient and give a written opinion on compliance with the due care criteria—and the administration of the medical treatment required to end the patient’s life or assist in suicide (Miller and Kim 2017).

In the Netherlands, review mechanisms entrusted to special committees (Regionale Toetsingscommissies Euthanasie, RTE) are also established: these committees are responsible for retrospectively evaluating whether physicians have met the applicable criteria. To ensure transparency, summaries of Dutch RTE decisions for cases of due care not met (DCNM) are available to the public.

It goes without say that the application of substantive criteria requires a diagnostic and decision-making act by professionals, while procedural criteria do not require extensive interpretation and are more automatic to perform. Nevertheless, an adequate definition of both is required in order to protect all the subjects involved. More specifically, the decision-making process regarding “unbearable and hopeless suffering” and “voluntary and thoughtful request” can be controversial, and there may be a difference of opinion between independent physicians (Buiting, et al. 2008). Professionals may approach the verification of the substantive criteria differently and find themselves faced with situations of uncertainty and ethical dilemmas. For this reason, and for the protection of all those involved, it is important that adequate verification methods be discussed and shared (Orentlicher, et al. 2016).

In Italy, there is currently no effective law on euthanasia or physician-assisted suicide, which are punishable under articles 579 (murder of a consenting person) and 580 (instigation or aiding of suicide) of the Italian Criminal Code. Recently, however, the Italian Constitutional Court (the “Court”) has been urged to rule in particular on the legitimacy of article 580 in certain circumstances of aiding suicide. The purpose of this contribution is to develop a reflection on the role that ethics committees, which, as discussed below, have been appointed by the Court, can possibly assume in general safeguard mechanisms. It is beyond the scope of the paper to consider the criteria or general conditions for access to assisted suicide practices or to discuss the contents of proposed laws.

## Italian Constitutional Court Decision No. 242 of 2019

Following specific petitions, starting with Order No. 207 in 2018, the Court have identified a series of circumstances in which criminal prosecution for assisting suicide, as established in Article 580 of the Criminal Code, might conflict with certain constitutional precepts. As the Italian Parliament had failed in the interim to enact a legislative act to regulate the matter, the 2018 order was followed in 2019 by a declaration of unconstitutionality of article 580 insofar as it prohibit physician-assisted death for a patient kept alive by life-sustaining treatments and affected by an irreversible medical condition that is a source of intolerable physical or mental suffering but who is fully capable of making free and conscious decisions.

Incidentally, and without being able to conduct an in-depth legal examination, it is worth mentioning some other examples of euthanasia and assisted suicide laws developed following the involvement of the Higher Courts. In 2015, in *Carter v. Canada*, the Supreme Court of Canada ruled that criminal laws prohibiting assistance in dying restrict certain personal rights enshrined in the Canadian Charter of Rights and Freedoms. The Court declared that

… s. 241(b) and s. 14 of the Criminal Code are void insofar as they prohibit physician-assisted death for a competent adult person who (1) clearly consents to the termination of life; and (2) has a grievous and irremediable medical condition (including an illness, disease or disability) that causes enduring suffering that is intolerable to the individual in the circumstances of his or her condition …

and suspended the declaration of invalidity for twelve months to give Parliament and provincial legislatures time to respond. In 2016, the Canadian Parliament passed a law to legalize and regulate euthanasia. In the wake of this case, in 2015 a plaintiff appealed to the New Zealand High Court seeking a declaration that the offense provisions of the Crimes Act are inconsistent with rights guaranteed by the New Zealand Bill of Rights Act 1990 (NZBORA). Here, the outcome was different. The court refused to make the statements requested by the plaintiff because, according to the judges, criminal law statements calling for changes in the effect of the offense provisions of the Crimes Act can only be made by Parliament. Quoting the judgment: “… The complex legal, philosophical, moral and clinical issues raised by Ms. Seales’ proceedings can only be addressed by Parliament passing legislation to amend the effect of the Crimes Act. …” In 2019, a law referred to as “The End of Life Choice Act” made assisted dying legal in New Zealand.

Italian Constitutional Court Judgement No 242 of 25 September 2019 established that an individual who, under specific circumstances, has facilitated the implementation of an independent and freely-formed resolve to commit suicide by another individual is exempt from criminal liability (Italian Constitutional Court 2019; Turillazzi, et al. 2021; Delbon, et al. 2021). The ruling refers specifically to the situation of a patient

… who is being kept alive by life-sustaining treatments and is suffering from an irreversible condition that is a source of intolerable physical or mental suffering but who is fully capable of taking free and informed decisions, provided that those conditions and the procedures for implementation have been verified by a public entity within the national health service, following issue of an opinion by the ethics committees with geographical jurisdiction …

As a result of this decision, which is self-enforceable, a number of citizens have submitted assisted suicide requests to the National Health Service, which is unable to respond adequately mainly due to the absence of common referral procedures. More specifically, there is no defined procedure regarding the drugs and the methods of administration to be used in order to guarantee the safety of and absence of discomfort for the patient.

As at July 2022, access to PAS had been granted for one Italian citizen, FC. As a result of his requests, FC was contacted by the Regional Health Service to establish a calendar of appointments with multidisciplinary specialists to verify compliance with the necessary conditions, after which the opinion of the Ethics Committee (EC) was sought. The Ethics Committee issued an opinion in which it confirmed the satisfaction of all but one of the conditions established with the Court’s ruling, as it did not express an opinion on the adequacy of the lethal drug and on the methods of administration proposed, considering the report to be insufficiently detailed and motivated. The EC pointed out that it is not its responsibility to indicate an alternative method. Following this uncertainty, the patient took legal action against the hospital in order to gain rapid access to the procedure. He eventually received a “Report from the multidisciplinary technical team on the selected modality, method and drug,” which served as the last missing step in order to fulfil his request.

Another patient, FR, chose disconnection from life support devices and deep palliative sedation because of the long waiting times for a response from the local health authority.

As this paper is being written, a third case is ongoing: that of a patient named A., who suffers from permanent quadriplegia following a road accident, has no cognitive impairment, and is completely dependent on a bladder catheter and on manual manoeuvres for defecation. After encountering resistance from the public health facility, A. appealed to the court, which ordered the Local Health Authority to: a) verify the existence of the conditions referred to in Constitutional Court Decision no. 242/2019 for the purpose of non-punishability of aiding of suicide practiced on his/her behalf by a third party; b) verify the effectiveness of the modality, in particular with regard to the identified drug. In compliance with the court ruling, the Health Authority set up an interdisciplinary commission to carry out the checks referred to in letters (a) and (b) and sent the final report to the competent Ethics Committee.

It should be noted that the report substantially confirmed the presence of all the conditions established by the Court. More specifically, it affirmed that the patient is “in a situation of intolerable psychological suffering” and stated:

(…) on the basis of the findings of the investigations it is possible to provide the Ethics Committee with the necessary elements to express an opinion on the basis of which the committee will respond to the Judge of the Court …

This notwithstanding, the Ethics Committee issued a very detailed report ending with the expression of an unfavourable opinion. The main rationale expressed in support of the negative response concerns the fact that, in the opinion of the Committee, the patient could be offered palliative care and interventions that might contribute to improving his self-sufficiency and quality of life. The Committee affirmed that this is the approach to be adopted in this specific case and declared that the palliative care proposed had been deficient. The stalemate generated by the conflicting opinions on an extremely complicated issue such as psychological suffering is considerable and is likely to generate further legal judgment.

## Discussion

Despite being immediately effective, Italian Constitutional Court Decision no. 242/2019 does not impose the healthcare facility’s duty to provide a service but merely the verification of the patient’s condition with the opinion of the geographically competent ethics committee. The analysis of the aforementioned cases clearly brings to light several critical issues. First of all, there is an overlap in the responsibility of two distinct subjects, the public National Health Service facility and the geographically competent committee. Both must carry out an assessment, the public healthcare facility must verify compliance with the legal requirements and the ethics committee must express an opinion; however, the form and specific subject-matter of this opinion are not clearly defined.

In the first case cited, that of FC, the territorial ethics committee issued an assessment based on the documentation and the collegial report received from the healthcare facility. The intervention of the committee took the form of a “second consultation”: the regional health service carried out the necessary checks on the patient and the ethics committee confirmed the satisfaction of the required conditions. This appears to be the same action performed twice, a double check; however, the Court does not seem to ask the ethics committee to verify the clinical condition of the patient, a task which is entrusted to the healthcare facility.

In the third case, that of A., on the other hand, the ethics committee commented on palliative care approaches and family and home care support, expressing a negative opinion on the request and referring the question back to the facility. Since the opinion of the ethics committee is not binding and given the positive verification of the conditions established by the Constitutional Court, it is likely that this patient will be able to proceed with his intent and obtain non-punishable aiding of suicide. The ambiguity of the procedural significance of this geographically-competent ethics committee opinion and its shortcomings as a mechanism for protecting the interested parties emerge clearly in this case.

In the current Italian legislative framework, there are forty Territorial Ethics Committees “CETs” and three National Ethics Committees “CENs,” which are mainly responsible for evaluating clinical trials and investigations with medical devices. As provided by the Decree of January 30, 2023, they can also perform advisory functions in relation to ethical issues related to research and clinical care activities. These organs are comparable to Research Ethics Committees (RECs) or Institutional Review Boards (IRBs), which apply, in Europe and the United States, respectively, with primary responsibility for reviewing clinical research protocols.

Requests for assisted suicide can originate from very demanding clinical cases. Research Ethics Committees do not meet patients and do not have a realistic chance to do so as a collegial body, so they do not seem to be in the best position to evaluate, for example, the patient’s decision-making capacity. It is not mandatory to include a psychiatrist or a physician experienced in palliative medicine in the composition of such committees. The specific reference of the Court’s 2019 Decision No. 242 appears to be to these bodies.

In Italy, alongside the CETs and CENs, there is also a network of different Committees that deal with ethical consultations. They have different names in the different regions of the country, such as Ethics Committee for Healthcare, Ethics Group, and Clinical Practice Ethics Committee (De Panfilis, et al. 2019; Picozzi and Gasparetto 2020). In their daily practice, they perform various functions, such as promoting the humanization of personal care, respect, and dignity, examining the ethical aspects associated with the planning and allocation of resources, service organization decisions and developing education and awareness initiatives for healthcare professionals and citizens. They can also be called upon as an advisory body with respect to complex clinical cases that pose ethical dilemmas.

Following the Court Decision no. 242, at the request of the Ministry of Health, some regions identified these Clinical Practice Ethics Committee as the bodies eligible for examining requests for assisted suicide (Petrini 2020). Recently, however, the Italian National Bioethics Committee, which performs both advisory functions to the government, parliament, and other institutions, referred to Territorial Ethics Committees (CETs) as the bodies “competent to express an opinion on assisted suicide” (Italian National Bioethics Committee 2023).

It is worth dwelling briefly on the meaning of an ethical consultation. It is not easy to define what a consultation process is, indeed there may be several ways to describe and conceive it. It is also not easy to answer the question: “what makes a case moral?” The question can be answered, for example, by stating that a case is moral when it involves issues relating to a patient’s self-sufficiency or competence in the informed consent process or end-of-life choices (Pedersen et al. 2010). Every moral act is such in relation to the good or evil of the action itself. It is possible to evaluate a clinical act with regard to i) to the agent’s intention, ii) to the scientific quality or adherence to the scientific gold standard, and iii) to compliance with the law. It is beyond the scope of this article to enter into the specific nuances of the meaning of the *morality* or *ethics* of a medical act or to analyse the methodology of ethical evaluation (Tambone and Ghilardi 2016). However, it is useful to clarify the terminology used. In this paper, ethical evaluation in the context of a clinical case means the activity of asking questions, scrutinizing, and discussing a situation that poses uncertainties, in order to identify the best course of action. This evaluation aims to find a solution in order to act in the best possible way having weighed up all the factors involved. Clinical ethics support, an activity of the aforementioned Clinical Practice Ethics Committees, begins when a professional encounters a problem with a practice and doubts the right course of action. A good example are the ethical dilemmas that can arise in healthcare facilities associated with respect for the patient’s self-sufficiency on the one hand and active intervention for the patient’s well-being on the other (Hartman, et al. 2019). The consultation arises from a spontaneous request associated with a need and cannot be imposed. No such ethical consultation for clinical practice can be imposed on a physician regarding a request for assisted suicide unless the physician requests it him/herself. If a physician is not uncertain about the situation in which he/she is about to act, for example, he/she undoubtedly believes that all the conditions required by law are met, he/she will not need any ethical consultation. This is because ethical consultation, by its very nature, is not a control mechanism, rather a support mechanism for complex decisions. Indeed, it is not binding and does not entail legal responsibility on the committee’s part.

Current PAS laws usually include advisory mechanisms but with a different meaning; neither elsewhere in Europe nor in the United States is there any involvement of ethics committees for clinical practice. The “consultant” is usually a specifically trained second physician who must consult with the patient and state in writing that the treating physician’s judgment has been formulated with due diligence. This is to ensure that the doctor who intends to perform the procedure has not overlooked anything (Ron, et. al 2012). The consultation of a second doctor in requests for euthanasia has the purpose of providing a control mechanism, and of monitoring and safeguarding the quality of the practice. Nevertheless, the consultation is generally not binding and the final legal responsibility lies with the physician performing the assisted suicide, who can, as a matter of fact, decide not to follow the advice, provided the reasons for this decision are stated.

A second point of interest for further research concerns precisely the role of the physician. The reference here is practical and legal responsibility rather than moral responsibility, a broad theoretical topic that is outside the scope of this discussion. In the current Italian scenario, the patient must apply not to a doctor but to the public facility to carry out the verification of the requirements. Such a facility may not know the patient at all. The individual must then independently find a doctor willing to support him/her in his intent of death. The process is therefore highly bureaucratized, fragmented, managed by the courts, to which people turn for quick answers, and outside the scope of a doctor–patient relationship of trust and care. Individual requests for medical assistance for suicide are complex in origin and require respect, careful attention, and open and sensitive communication in the clinical setting (Materstvedt, et al. 2003). Which physician should provide information to the patient (diagnosis, prognosis, treatment options), discuss eligibility, advise families, prescribe the lethal drug, and offer support during administration? Are they ready to participate in the process? The absence of guidelines places the physician in a difficult position and makes the whole procedure unclear. As noted, although there is an abundance of literature discussing the ethical issues, there is relatively little literature addressing the medical aspects of providing aid in dying. In countries where legislation is in place, the roles and responsibility of healthcare professionals and other supporting non-governmental associations is well defined (Stephen 2007). A general practitioner with whom the patient has a personal and long-standing relationship is in a better position to judge whether a patient meets the required criteria for care. However, physicians are burdened with decisions that, particularly in complex cases, can go beyond normal medical practice and constitute a moral dilemma (Snijdewind, et al. 2018). Their role must be carefully determined in order to protect both sides of the relationship—doctor and patient. Some scholars have also proposed the establishment of systems that remove the physician from direct involvement in the process. In this case, the physician would only be in charge of diagnosis and prognosis, while a central mechanism would have to confirm the authenticity and eligibility of patient requests, dispense drugs, and monitor demand and use (Prokopetz and Lehmann 2012). Given their pivotal role, in-depth analysis and empirical research into physicians’ attitudes are critical when jurisdictions move from a lawless to an enforcement context. The design of legislative models must take into account the organizational and cultural context and the concerns of physicians (Rutherford, et al. 2021; Zworth, et al. 2020).

## Conclusion

The act of assisting suicide involves situations of great vulnerability and concerns the heart of the ethics of the medical profession. Constitutional Court Decision No. 242 of 2019 involved territorial ethics committees, which are those primarily responsible for evaluating clinical trials and investigations with medical devices, without specifying in detail what they are called upon to do. Precisely, the Court states:

The fragility of the value at stake requires the intervention of a third collegiate body with the appropriate expertise, which can ensure the protection of situations of particular vulnerability. Pending the intervention of the legislature, this task is entrusted to the geographically-competent ethics committees.

The involvement of such committees is based on the intention to protect subjects in a situation of legislative and procedural vacuum, yet the function they are supposed to perform seems uncertain. In this contribution, it has been pointed out that an advisory function on ethical dilemmas is very different from a control and verification function and that, if the roles are unclear, stalemates are likely to occur. Ethical analysis and evaluation should be applied upstream to guide legislative changes, to conduct a thorough analysis of possible trade-offs between individual freedom and the safety of society, and to balance the needs of all actors in the process. The values at stake must be made explicit, and the best scientific evidence must be sought and applied. When considering a PAS law, the clarification of responsibilities, access criteria, and safeguards is imperative. The most difficult task for jurisdictions that have legalized or intend to legalize assisted dying is to ensure that the safeguards identified achieve their intended purpose (Sleeman and Owen 2021). In addition to establishing access criteria, it is equally essential to discuss in detail how the criteria will be verified. Therefore, it is a priority in Italy to develop a research agenda that includes all of these issues. The involvement of ethics committees seems ambiguous in the context of a PAS law, as it would be in any law establishing the conditions of access to medical health services, unless it is intended solely as a support tool for physicians dealing with particularly problematic cases, the latter function more proper to clinical ethics committees.
